# Saponins-Containing Plants of the Genera *Gypsophila* L. and *Acanthophyllum* C. A. Mey. (Caryophyllaceae) from Southeastern Kazakhstan: Root Morphometry, Saponins Content and Antimicrobial Activity of Extracts

**DOI:** 10.3390/plants15193077

**Published:** 2026-10-08

**Authors:** Valentina Mursaliyeva, Nadezhda Gemejiyeva, Balaussa Sarsenbek, Gulnara Dzhakibayeva, Tlek Mukhanov, Ramazan Mammadov

**Affiliations:** 1 Institute of Plant Biology and Biotechnology, 45 Timiryazev St., Almaty 050040, Kazakhstan; 2 Institute of Botany and Phytointroduction, 36 D Timiryazev St., Almaty 050040, Kazakhstan; 3 Research and Production Center of Microbiology and Virology, 105 Bogenbai batyr St., Almaty 050010, Kazakhstan; 4 Department of Biology and Ecology, Faculty of Nature and Technology, University of Odlar Yurdu, Baky AZ1072, Azerbaijan

**Keywords:** *Gypsophila paniculata*, *Gypsophila perfoliata*, Acanthophyllum pungens, root, phase of vegetation, total saponins, antimicrobial activity

## Abstract

The relationship between root morphometry, growth stages, and total saponin content (TS) by a vanillin-sulfuric acid method in the wild-growing *Gypsophila paniculata* (*n* 43), *G. perfoliata* (*n* 35), and *Acanthophyllum pungens* (*n* 47) from southeastern Kazakhstan was studied and a preliminary assessment of their inhibitory activity against *Bacillus* sp., *Erwinia amylovora*, *Agrobacterium* sp., *Fusarium* sp., and *Aspergillus niger* was conducted by the disk-diffusion method. ANOVA and correlation analysis of root parameters and TS content during the vegetation revealed: *G. perfoliata* exhibited consistent TS level and similar root indices during all phases; *G. paniculata* showed maximum TS at the flowering coinciding with minimal root diameter values; *A. pungens*—high TS accumulation and high root growth indices at the end of the vegetation. The differences among species indicate varying root growth dynamics during the growing season, which influence the TS accumulation. The variability in morphometry and TS at separately phase of growth may be associated with intrapopulation heterogeneity. The antibacterial activity of the extracts (10:1) varied depending on the target bacterial species and the extract dose. *Gypsophila* extracts did not differ in their activity against G+ *Bacillus* sp., *G– Er. amylovora*, and *Fusarium* sp. The *A. pungens* activity range additionally included *G-Agrobacterium* sp. and *Aspergillus niger*. These results may serve as a basis for further study of these species as promising sources of saponins with antimicrobial activity.

## 1. Introduction

Plants of the Caryophyllaceae family are valuable medicinal and industrial crops, many of which are widely used in traditional medicine, the official pharmaceuticals, the food industry, cosmeceuticals, the production of detergents, and other fields. Several reviews discuss ethnomedicinal uses, pharmacological studies [1,2], surfactant properties [3], food functionality and other application prospects of saponins-containing plants [4,5]. The main secondary metabolites of plants in the Caryophyllaceae family are triterpene saponins [6,7]. As plant secondary metabolites, saponins perform a variety of physiological functions, including protecting the plant against a wide range of pathogenic microorganisms, harmful insects, and herbivorous animals [8,9]. The family Caryophyllaceae is represented by 224 species in the flora of Kazakhstan, of which saponins have been identified in 97 species [10,11]. Species of the genera *Gypsophila* L. and *Acanthophyllum* C. A. Mey. occupy a special place due to the high content of triterpene saponins in their underground parts, reaching 30% of saponins in dry matter [2,12,13,14]. An analysis of recent studies indicates that *Gypsophila* species are currently considered among the most promising natural sources of new antimicrobial pharmaceuticals [15,16,17] and potential biopesticides for protecting plants from various pathogens and controlling insect pests [18,19,20,21,22,23]. The most noticeable activity of saponins extracts is observed against the Gram-positive bacteria *Staphylococcus aureus*, *Bacillus subtilis*, and *Bacillus cereus* [24]. The primary mechanism of saponins action is believed to be the disruption of the cytoplasmic membrane, accompanied by an increase in its permeability and subsequent cell death [25]. An important advantage of saponins is their ability to enhance the effects of antibiotics and inhibit the formation of bacterial biofilm, as demonstrated in several research [16,26,27]. Several studies have shown that saponins content is determined not only by the plant’s genotype but also depends on its age and stage of ontogenesis, growing conditions, and root morphometry [28,29,30]. The accumulation of secondary metabolites is a dynamic process closely linked to the seasonal redistribution of carbon between aboveground and belowground organs [31,32]. However, for species *Gypsophila* and *Acanthophyllum* and their natural populations in southeastern Kazakhstan the effects of the ontogenetic and vegetative phases, plant age, and root morphometric parameters on saponin accumulation remain unexplored. The quality of the raw plant material—including its size, weight, and harvest timing, considering the seasonal dynamics of secondary metabolite accumulation—is of crucial importance for the practical use of saponins-bearing plants.

The aim of this study was to investigate the relationship between certain root morphometric parameters, the vegetative phase, and total saponin content, and to conduct a preliminary assessment of the antimicrobial activity of total extracts from *Gypsophila paniculata* L., *Gypsophila perfoliata* L., and *Acanthophyllum pungens* (Bunge) Boiss., collected from natural populations in the desert region of southeastern Kazakhstan.

## 2. Results

### 2.1. Collection and Characterization of Raw Material Parameters

The underground parts of wild plants were dug up from natural populations in the Almaty Region between May and September at various phase of their growth. The number of raw material sets was: 7 for *G. paniculata*, 5 for *G. perfoliata*, and 5 for *A. pungens* (Table 1).

The data presented in Table 1 show that the timing of the growing season and its duration varied among species in terms of months and the length of individual phases. For example, the growing season for *G. paniculata* in April began one month earlier than the onset of growth for *G. perfoliata* and *A. pungens*, which began their growing season in early May. The budding, flowering, and fruiting stages in *G. paniculata* and *A. pungens* occurred over a relatively short period during the month of May. *G. paniculata* plants formed buds and bloomed throughout May, and by June, the aboveground parts of the plants had already ceased growing. *A. pungens* had the shortest growing season, with new shoots emerging from last year’s shrub in early May and the above-ground parts of the plants (“tumbleweed”) drying out by the first half of June. *G. perfoliata* plants had a fairly long growing season, lasting from May through September. During this period, from May through June, new shoots were observed to emerge, and the aboveground parts of *G. perfoliata* plants grew actively. Bud formation was observed in the second half of July; flowering and fruiting occurred throughout August; and the gradual drying out and die-off of the aboveground parts at the end of the growing season began in September.

**Figure 1:**
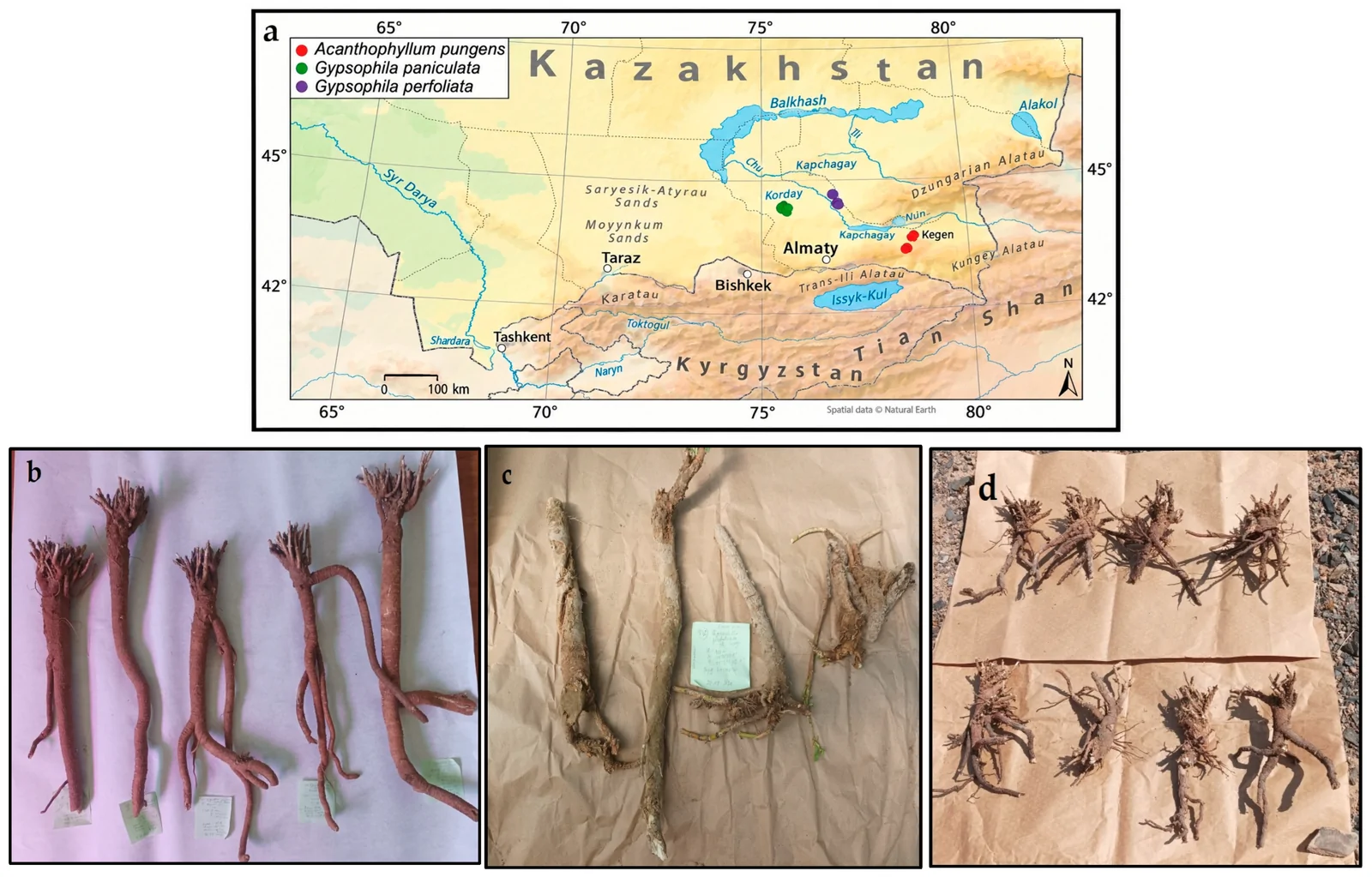
The location of wild populations in the surveyed area of southeastern Kazakhstan (**a**) and samples of collected roots: *Gypsophila paniculata* (**b**), *G. perfoliata* (**c**), *and Acanthophyllum pungens* (**d**).

The location of wild populations in the surveyed area of southeastern Kazakhstan is presented in Figure 1a. The samples of collected roots of *G. paniculata*, *G. perfoliata*, and *A. pungens* are shown in Figure 1b, Figure 1c and Figure 1d, respectively.

**Figure 2:**
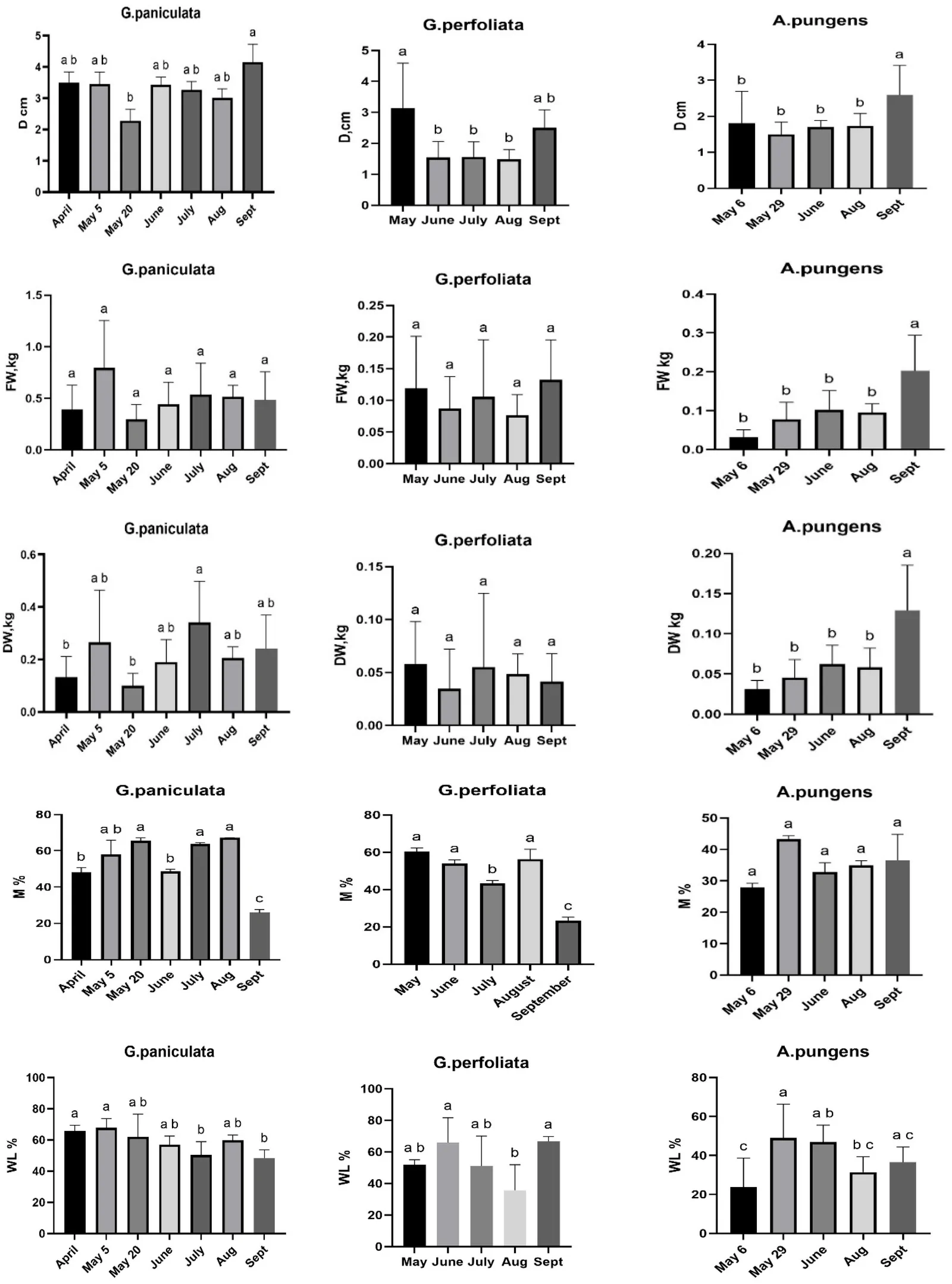
Diameter (D cm), fresh weight (FW kg), dry weight (DW kg), moisture (M %) and weight loss (WL%) of root samples of *Gypsophila paniculata*, *G. perfoliata*, and *Acanthophyllum pungens* collected during the growing season from April to September. M ± SD. Significant differences are represented by different letters (a–c) *p* ≤ 0.05.

Summary mean data from the surveys on root diameter (D), fresh weight (FW), air-dry weight (DW), moisture content (M), and weight loss (WL) during drying of the raw material are presented in Table A1 and Figure 2.

Intraspecific and interspecific differences in these parameters were identified depending on the time of root harvest and the growth stage of the source plants. Freshly dug roots of *G. paniculata* were the largest (Figure 1b); the fresh weight (FW) of individual samples collected at different times ranged from a minimum of 0.3 kg to a maximum of 1.5 kg, and the dry weight (DW) ranged from 100 g to 500 g. The maximum diameter (D) of 4.15 ± 0.58 cm was observed in roots dug up in September toward the end of the growing season. The minimum D of 2.28 ± 0.37 cm was observed in roots collected in the second decade of May from flowering parent plants. The ANOVA results showed that *G. paniculata* samples differed by harvest time in terms of root D, though the difference was only marginally significant (F = 2.41, *n* = 43, *p* < 0.05); there were no significant differences in FW (F = 1.81, *p* > 0.05), but the differences in DW were highly significant, F = 3.44, *p* < 0.01. The average coefficient of variation was 26% for D and, on average, 50% for both FW and DW (Table A1).

The roots of *G. perfoliata* and *A. pungens* had smaller measurements compared to those of *G. paniculata* and did not differ significantly from one another (Figure 1c,d). However, the patterns of growth parameter dynamics during the growing season differed among the species. Root samples of *G. perfoliata* collected at the beginning of the growing season (in May) had the maximum morphometric values for this species: average D 3.13 ± 0.46 cm, FW 0.12 ± 0.03 kg, and DW 0.06 ± 0.01 kg. In subsequent collections, from June through August during the active growing season, bud formation, and flowering phases, root growth parameters were half as high; however, at the very end of the growing season (September), the parameters returned to the May values. ANOVA of the *G. perfoliata* data revealed statistically significant differences across harvest times only for D (F = 5.67, *n* = 35, *p* < 0.01); there were no significant differences in FW or DW (F = 0.60; F = 0.34, *p* > 0.05).

Root samples of *A. pungens* collected from May through early August did not differ in D, FW, or DW. The most distinctive characteristics, with the highest values, were observed in roots collected in September at the very end of the growing season: D 2.59 ± 0.26 cm, FW 0.20 ± 0.03 kg, and DW 0.13 ± 0.02 kg. ANOVA of the data on *A. pungens* harvest dates revealed: a statistically significant difference in D (F = 4.85, *n* = 47, *p* < 0.05), and highly significant differences in FW and DW, F = 14.18; F = 14.04, *n* = 47, *p* < 0.001, respectively. The root moisture content (M) of *G. paniculata* and *G. perfoliata* varied only slightly during the growing season and decreased by nearly half only at the end of the growing season. In contrast, the M of *A. pungens* fluctuated insignificantly across harvest dates (F = 2.08, *p* > 0.05) and remained at an average level throughout the entire growing season. Weight loss (WL) during drying of the raw material for all three species did not vary significantly by harvest date and averaged 56% for *G. paniculata* and *G. perfoliata* and 38% for *A. pungens* root samples. Table 2 presents the average values of root parameters for *G. paniculata*, *G. perfoliata*, and *A. pungens*, aggregated across all collections, for the entire growing season.

An analysis of the mean data presented in Table 2 shows that the root samples of *G. paniculata* significantly outperformed those of *G. perfoliata* and *A. pungens*, with the following mean root parameters: D 3.26 ± 0.14 cm, FW 0.49 ± 0.04 kg, DW of 0.23 ± 0.02 kg. The collected root samples of *G. perfoliata* and *A. pungens* did not differ significantly from one another in terms of D, FW, or DW. The *Gypsophila* root samples also did not differ in terms of average M (55.27 ± 3.45% and 47.03 ± 4.08%, respectively), whereas the M of *A. pungens* was 1.5 times lower, with a mean of 35.34 ± 1.90% (F = 9.50, *n* = 39, *p* < 0.01).

### 2.2. Saponins Content

Total saponin content, expressed as a percentage of absolutely dry weight (% a.d.w.) in crude root extracts from different harvests during the growing season of the studied species, are presented in Table A1.

Analysis of these data showed that the dynamics of TS in roots during the growing season differ among species. In the roots of *G. paniculata*, the initial saponins level at the start of the growing season—with a mean of 31.87 ± 4.59% a.d.w.—rose to 50.79 ± 8.58% a.d.w. during the budding phase, and then to a maximum (98.35 ± 2.47% a.d.w.) during flowering in late May. Starting in June, as the aboveground parts withered and the growing season came to an end, the TS in the roots decreased by half to its initial level and remained stable, reflecting the decrease in moisture content of the raw material at the end of the growing season. The average TS in the roots of *G. paniculata* over the entire growing season was 52.44 ± 3.94% on a completely dry raw material basis. ANOVA of the TS data for *G. paniculata* revealed highly significant differences among samples from different harvests (F = 14.94, *n* = 43, *p* < 0.001).

In the roots of *G. perfoliata*, the initial high TS of 65.54 ± 15.24% a.d.w. (May harvest) gradually decreased during the growing season to an average of 31.04 ± 4.70% a.d.w. on the budding-flowering phase (July–August) and to 48.97 ± 7.17% a.d.w. by the end of the growing season. At the same time, an ANOVA of the *G. perfoliata* data showed no statistically significant differences between harvests (F = 1.95, *n* = 35, *p* > 0.05); the average TS in the roots of *G. perfoliata* over the growing season was 46.37 ± 5.03% a.d.w.

*A. pungens* showed excellent dynamics: saponins levels varied only slightly at the beginning of the growing season and during flowering, ranging from 36.87 ± 5.22% a.d.w. to 26.70 ± 2.48% a.d.w., then decreased to 19.23 ± 1.48% a.d.w. in August, but in September, at the very end of the growing season, it rose to a maximum value of 66.18 ± 9.72% a.d.w. with a high level of significance (F = 11.62, *n* = 47, *p* < 0.001). The average TS level in the roots of *A. pungens* over the growing season was 36.99 ± 3.22% a.d.w.

**Figure 3:**
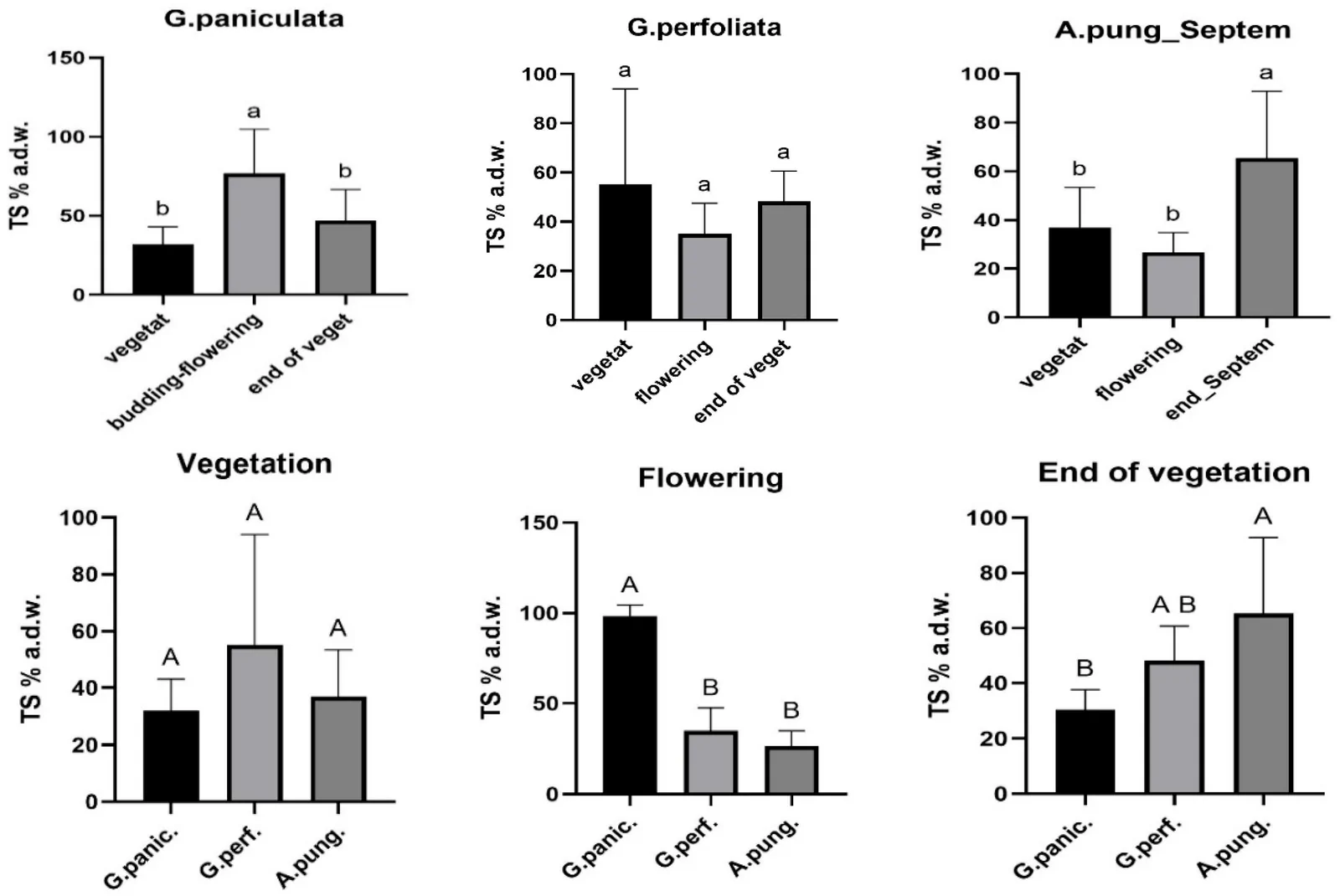
Total saponin content (% a.d.w.) in the roots of wild-growing *G. paniculata*, *G. perfoliata*, and *A. pungens* plants, depending on the growth stage. M ± SD. Significant differences are represented by different letters (a–b, the top row; A-B, the bottom row) *p* ≤ 0.05.

Table A2 and Figure 3 present summarized TS data by growth stage for a single species and grouped by specific stages (vegetation, flowering, and the end of the growing season) for all three species.

The data show that statistically significant differences in TS across growth stages were found in *G. paniculata* and *A. pungens* (F = 11.32, F = 11.56, *p* < 0.001). The maximum TS in the roots of *G. paniculata* was observed during the flowering stage, while for *A. pungens* it was observed at the very end of the growing season; for both species, this level was twice as high as that observed during other stages of the growing season (Figure 3).

A comparison of the species across individual growth phases revealed the following: during the vegetative stage, there were no differences between the species (F = 1.89, *n* = 33, *p* > 0.05); during the flowering stage, saponins levels were higher in *G. paniculata* (F = 25.30, *n* = 36, *p* < 0.001), and at the end of the growing season (September), *A. pungens* had higher saponins levels (F = 3.84, *n* = 19, *p* < 0.05).

**Figure 4:**
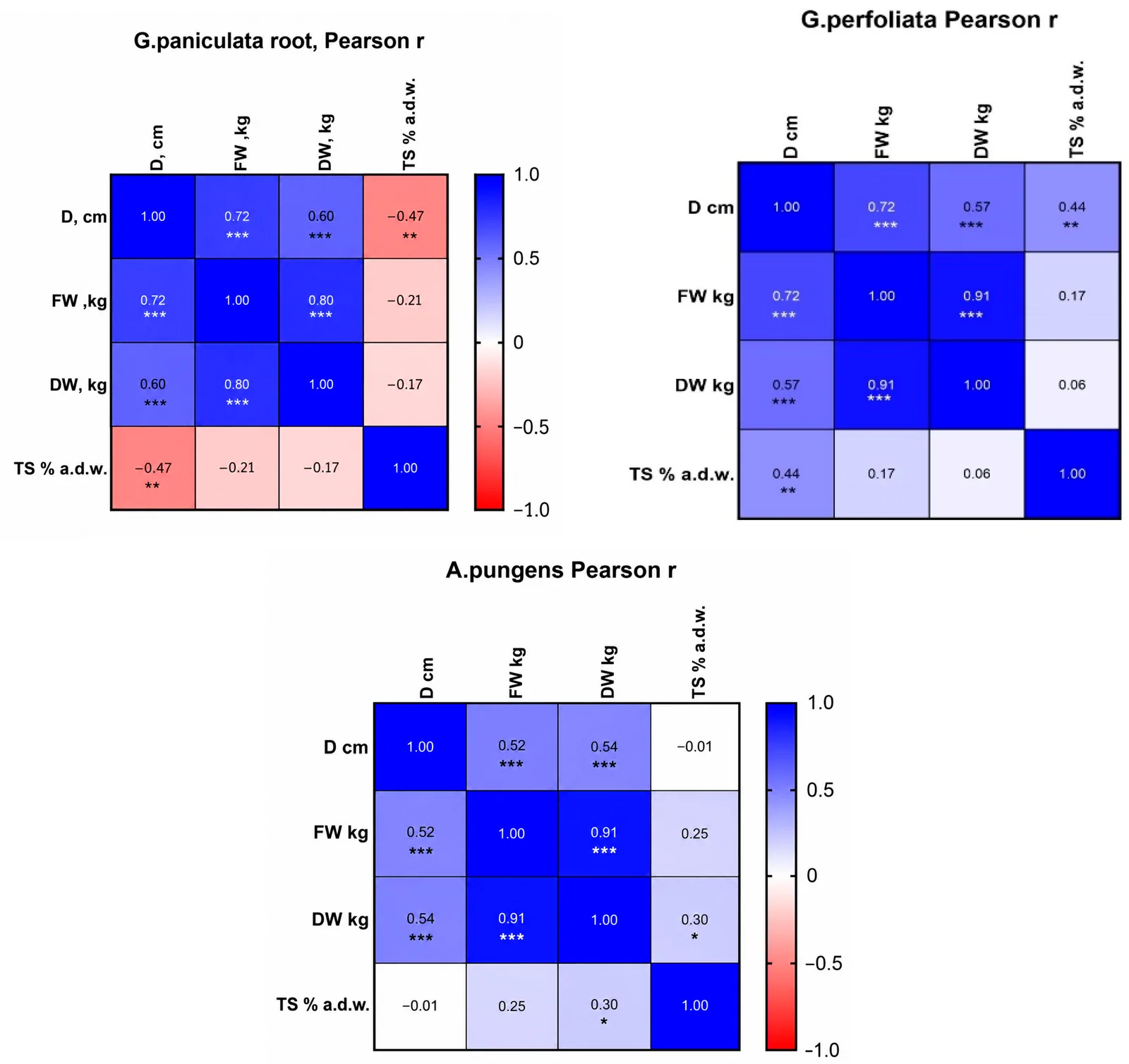
Correlation heatmap showing relationships between root parameters (diameter D, fresh weight FW, dry weight DW) and their total saponin content (TS) for *Gypsophila paniculata*, *Gypsophila perfoliata*, and *Acanthophyllum pungens*. Positive correlations are represented by increasing intensity, while negative correlations indicate inverse relationships between variables. * *p* ≤ 0.05: statistically significant; ** *p* ≤ 0.01: highly significant; *** *p* ≤ 0.001: very highly significant.

To identify relationships between the quantitative indicators of the roots, a correlation analysis was conducted using Pearson’s correlation coefficient r (Figure 4).

A correlation analysis of the data revealed statistically significant relationships between various root parameters, as well as between these parameters and total saponin content. A strong correlation was found between FW and DW in all species; *G. paniculata* r = 0.80, *G. perfoliata* and *A. pungens* r = 0.91. In *G. paniculata* and *G. perfoliata* significant correlation between D and FW (r = 0.60, r = 0.72, respectively), and between D and DW (r = 0.60, r = 0.57, respectively) were detected. FW and DW of *A. pungens* were correlated with D (r = 0.52, r = 0.54) and TS were moderate positive correlated with dry weight DW (r = 0.30, *p* < 0.05). The species *Gypsophila* differed diametrically regarding the relationship between TS and D: moderate positive correlation in *G. perfoliata* (r = 0.44), whereas *G. paniculata* showed a negative one (r = –0.47).

### 2.3. Antimicrobial Activity of Extracts

Varying degrees of sensitivity were observed in the test strains of bacteria—*Bacillus subtilis*, *Bacillus megatericum*, *Erwinia amylovora*, and *Agrobacterium* sp.—and fungal microorganisms—*Fusarium solani*, *Fusarium graminearum*, and *Aspergillus niger*—to various antibiotics and nystatin (Table 3).

**Figure 5:**
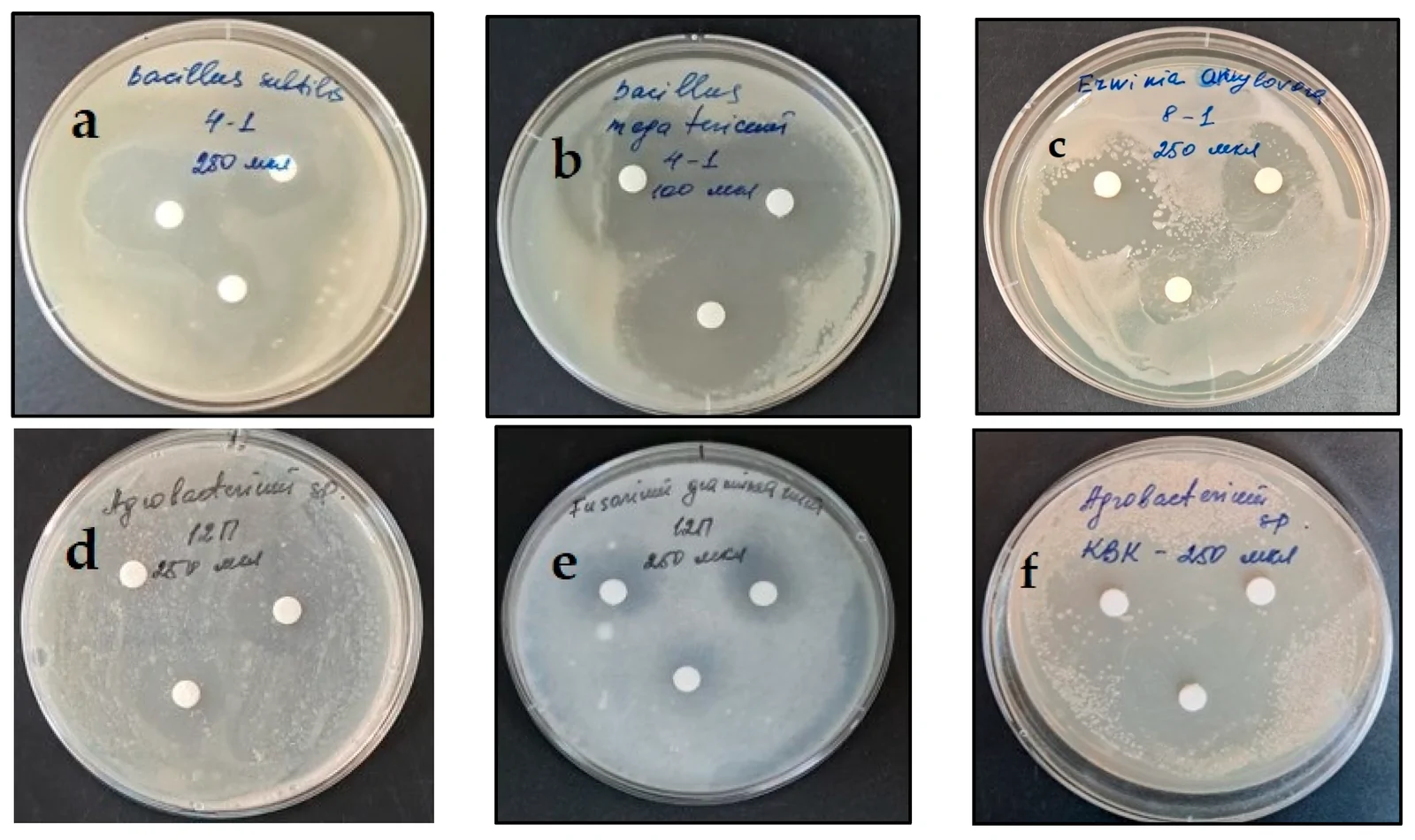
Growth-inhibiting activity of the total saponins extracts (10:1): *G. paniculata* to *Bacillus megatericum*, zone 11.00 mm at 250 µL (**a**); *G. paniculata* to *Bacillus megatericum*, zone 15.33 mm at 250 µL (**b**), *G. perfoliata* to *Erwinia amylovora*, zone 12.33 mm at 250 µL (**c**); *A. pungens* to *Agrobacterium* sp., zone 11.00 mm at 250 µL (**d**), *A. pungens* to *Fusarium graminearum*, zone 9.67 mm at 250 µL (**e**), inhibitory zone of 19.67 mm of commercial saponin from *Quillaja bark* (10:1) at 250 µL in a culture of *Agrobacterium* sp. (**f**).

The test strain of *B. megatericum* was susceptible to lincomycin and erythromycin (inhibition zones of 14.66 and 11.66 mm, respectively), but was resistant to ampicillin, with the lowest inhibition zone of 2 mm. *B. subtilis*, *Er. amylovora*, and *Agrobacterium* sp. were susceptible to all antibiotics, with inhibition zones ranging from 10 mm to 20 mm. The antifungal agent nystatin inhibited the growth of *Aspergillus niger* and *Fusarium graminearum* with inhibition zones of 10.66 mm and 14.33 mm, respectively. The *Fusarium solani* fungal culture was found to be resistant to nystatin, which did not inhibit the growth of the pathogen. The results of the evaluation of total saponin extracts from *G. paniculata*, *G. perfoliata*, and *A. pungens* (10:1), as well as the control, on the test cultures are presented in Table 4 and Table 5 and Figure 5.

In the control (70% ethanol), the inhibition zones for Gram-negative (G-) bacteria *Agrobacterium* sp. and *Er. amylovora* were 2.33 mm and 5.66 mm, respectively. The ethanol control did not inhibit the growth of Gram-positive (G+) bacteria *Bacillus* cultures. The growth-inhibiting activity of the test extracts varied depending on their dose and the bacterial species. None of the three extracts, at doses of 25 µL and 50 µL, inhibited the growth of *Bacillus* sp. Extracts of *G. paniculata* and *G. perfoliata* at 100 µL and 250 µL exhibited similar inhibitory activity against two G+ *Bacillus* species (Figure 5a,b), with inhibition zones ranging from 11 mm to 15 mm (F = 2.33, *n* = 12, *p* > 0.05; F = 0.51, *n* = 12, *p* > 0.05, respectively).

*Gypsophila* extracts had no effect on the growth of G– *Agrobacterium* sp. The growth-inhibiting effect of *Gypsophila* extracts on G– *Er. amylovora* varied depending on the applied dose: at 25 µL and 100 µL, the effect did not exceed that of the 70% ethanol control; at 50 µL and 250 µL, the maximum zone diameter had a mean value of 9.17 mm for *G. paniculata* and 10.83 mm for *G. perfoliata*. ANOVA revealed statistically significant differences in the data for *G. paniculata* (F = 25.84, *n* = 12, *p* < 0.001) and for *G. perfoliata* (F = 13.16, *n* = 12, *p* < 0.01). Significant differences in inhibition zones were observed between *Bacillus* and *Er. amylovora* cultures at the same dose of *G. paniculata* extract: at 100 µL, the zones measured 14.67 mm and 4 mm, respectively (F = 42.86, *n* = 9, *p* < 0.001); at a dose of 250 µL—*B. subtilis* 11 mm, *B*. *megatericum* 15.33 mm, *Er. amylovora* 9.33 mm (F = 5.89, *n* = 9, *p* < 0.05). The growth-inhibitory effect of the *G. perfoliata* extract was like that of the *G. paniculata* extract at a dose of 100 µL (F = 41.08, *n* = 9, *p* < 0.001). At a dose of 250 µL, the activity of the *G. perfoliata* extract did not differ significantly among the test cultures, with inhibition zones ranging from 12.33 mm for *Er. amylovora* (Figure 5c) to 14.00 mm–15.33 mm for *Bacillus* (F = 2.10, *n* = 9, *p* > 0.05).

Unlike the *Gypsophila*, the *A. pungens* extract inhibited the growth of G– *Agrobacterium* sp. (Figure 5d) at all tested doses, exceeding the ethanol control: 2-fold at 25 µL and 100 µL, and 5-fold at 50 µL and 250 µL. ANOVA revealed highly significant differences in the activity of *A. pungens* against *Agrobacterium* sp. (F = 51.07, *n* = 12, *p* < 0.001). Along with, the *A. pungens* extract inhibited the growth of *Er. amylovora* only at the maximum dose of 250 µL, with an inhibition zone of 11.67 ± 1.67 mm. With regard to G+ *Bacillus* cultures, the activity of the *A. pungens* extract did not differ from that of the *Gypsophilla* extracts: effective doses were 100 µL and 250 µL, with inhibition zones ranging from 11 mm to 15 mm. ANOVA did not reveal any significant differences in the data for *A. pungens* relative to *Bacillus* and *Er. amylovora* cultures (F = 2.08, *n* = 15, *p* > 0.05). The inhibitory effect of the *A. pungens* extract at a dose of 100 µL was significantly greater against G+ *Bacillus* cultures than against G– *Agrobacterium* sp. (F = 42.86, *n* = 9, *p* < 0.001). A higher dose of the extract (250 µL) showed activity against all four test cultures, with no significant differences in the inhibition zones (F = 2.29, *n* = 12, *p* > 0.05).

Two-way ANOVA (including zero values where no activity was observed) on the antibacterial activity of the extracts revealed a significant effect of extract dose, type of bacterial test culture, and their interaction on the inhibition zone diameter (Table A3). The growth-inhibiting effect of extracts from *G. paniculata*, *G. perfoliata*, and *A. pungens* is highly likely to be dose-dependent, with contribution rates of 36.76%, 37.94%, and 49.45%, respectively. The effect of test culture type on the outcome measure was also significant, with contribution rates of 24.03% for *G. paniculata*, 24.76% for *G. perfoliata*, and 10.33% for *A. pungens*. The combined effect of the test culture and extract dose on the performance indicator did not differ significantly among species: 36.87% for *A. pungens*, 35.24% for *G. perfoliata*, and 36.87% for *G. paniculata*.

The antifungal activity of the extracts varied depending on the source plant species, the extract dose, and the test fungal species (Table 5).

*G. paniculata* extract inhibited the growth of two *Fusarium* species at 50 µL, 100 µL, and 250 µL, with a maximum inhibition zone of 8.00 mm at the minimum dose. However, ANOVA did not reveal any significant differences in the data for *G. paniculata* regarding nystatin-resistant *F. solani* (F = 4.59, *n* = 9, *p* > 0.05) or regarding nystatin-susceptible *F. graminearum* (F = 1.33, *n* = 9, *p* > 0.05). With regard to *A. niger*, the activity of the *G. paniculata* extract at the same dose of 50 µL was lower, with a maximum inhibition zone of 4.33 mm. ANOVA did not reveal any significant differences in the inhibition zone among the three test cultures (F = 1.79, *n* = 9, *p* > 0.05). When the dose was increased to 100 µL and 250 µL, the activity of the *G. paniculata* extract decreased significantly or was absent. At a dose of 100 µL, significant differences in zone were observed among the cultures (F = 8.81, *n* = 9, *p* < 0.05). The growth-inhibiting effect of *G. paniculata* extract at 100 μL was slightly higher against *F. solani*. Two-way ANOVA of the all applied doses (25 µL, 50 µL, 100 µL, and 250 µL) across all cultures showed a predominant effect of the test-culture factor (53.67%), a lesser contribution from the dose factor (19.02%) and their interaction (11.47%) on the zone size (Table A4).

The maximum activity of *G. perfoliata* extracts against *F. solani* and *F. graminearum* (zones of 9.33 mm and 7.67 mm, respectively) was observed at 50 µL. When the dose was increased to 100 µL or 250 µL, the activity decreased, zone of 2.67 mm and 3.67 mm for *F. solani*, respectively, and 4.33 mm at 250 µL for *F. graminearum* (Table 5). ANOVA revealed highly significant differences in the data of *G. perfoliata* against *F. solani* (F = 38.78, *n* = 9, *p* < 0.001). With regard to *A. niger*, the activity of the *G. perfoliata* extract was observed only at 50 µL, with a maximum inhibition zone of 6.67 mm. The growth-inhibiting effect of *G. perfoliata* extract at 50 μL was slightly higher against *F. solani* (F = 8.17, *n* = 9, *p* < 0.05). Two-way ANOVA of *G. perfoliata* across dose levels (25 µL, 50 µL, 100 µL, and 250 µL) showed a predominant effect of the dose factor (83.65%), a lesser significance of the test-culture factor (7.64%) and their interaction (6.73%) on the zone size (Table A4).

The *A. pungens* extract exhibited activity against all three-fungus, *F. solani*, *A*. *niger*, and *F. graminearum* (Figure 5e) at 50 µL and 250 µL. Maximum activity (zone from 8.67 mm to 10 mm) was observed at a dose of 250 µL without significant differences among the test fungal cultures (F = 0.37, *n* = 9, *p* > 0.05). Significant differences among the test fungus were observed at 50 µL (F = 13.59, *n* = 9, *p* < 0.01) and the growth-inhibitory activity of *A. pungens* against *F. graminearum* was, on average, 1.6 times greater than its activity against *F. solani* and *A. niger*. Increasing the extract dose of the *A. pungens* from 50 µL to 250 µL did not result in an increase in the inhibition zone for *F. graminearum*, as was observed for *F. solani* and *A. niger*. Two-way ANOVA of variance for the whole test doses (25 µL, 50 µL, 100 µL, 250 µL) revealed that the dose of *A. pungens* extract had a significant effect (89.65%) on the inhibition zone size. The combined effect of the test culture and the extract dose on the outcome did not exceed 5.27% (Table A4).

A commercial saponins preparation derived from the bark of the soapberry tree, *Quillaja saponaria* (Cas. No. 8047-15-2), was used as a comparative control. It was found that the commercial saponins preparation, at 250 µL, exhibited activity against two G+ *Bacillus* species c with zone 11.67 mm and 13.00 mm, while the maximum zone of 19.67 mm was observed against *Agrobacterium* sp. (Figure 5f) with high significance (F = 99.20, *n* = 9, *p* < 0.001). The zones of growth inhibition for *F. solani* and *As. niger* did not differ from one another, with an average of approximately 10 mm. *Q. saponaria* exhibited high activity against *F. graminearum*, with a maximum inhibition zone of 15.10 mm. The antifungal activity of the *Quillaja saponaria* preparation varied significantly depending on the test fungal cultures (F = 49.41, *n* = 9, *p* < 0.001).

**Figure 6:**
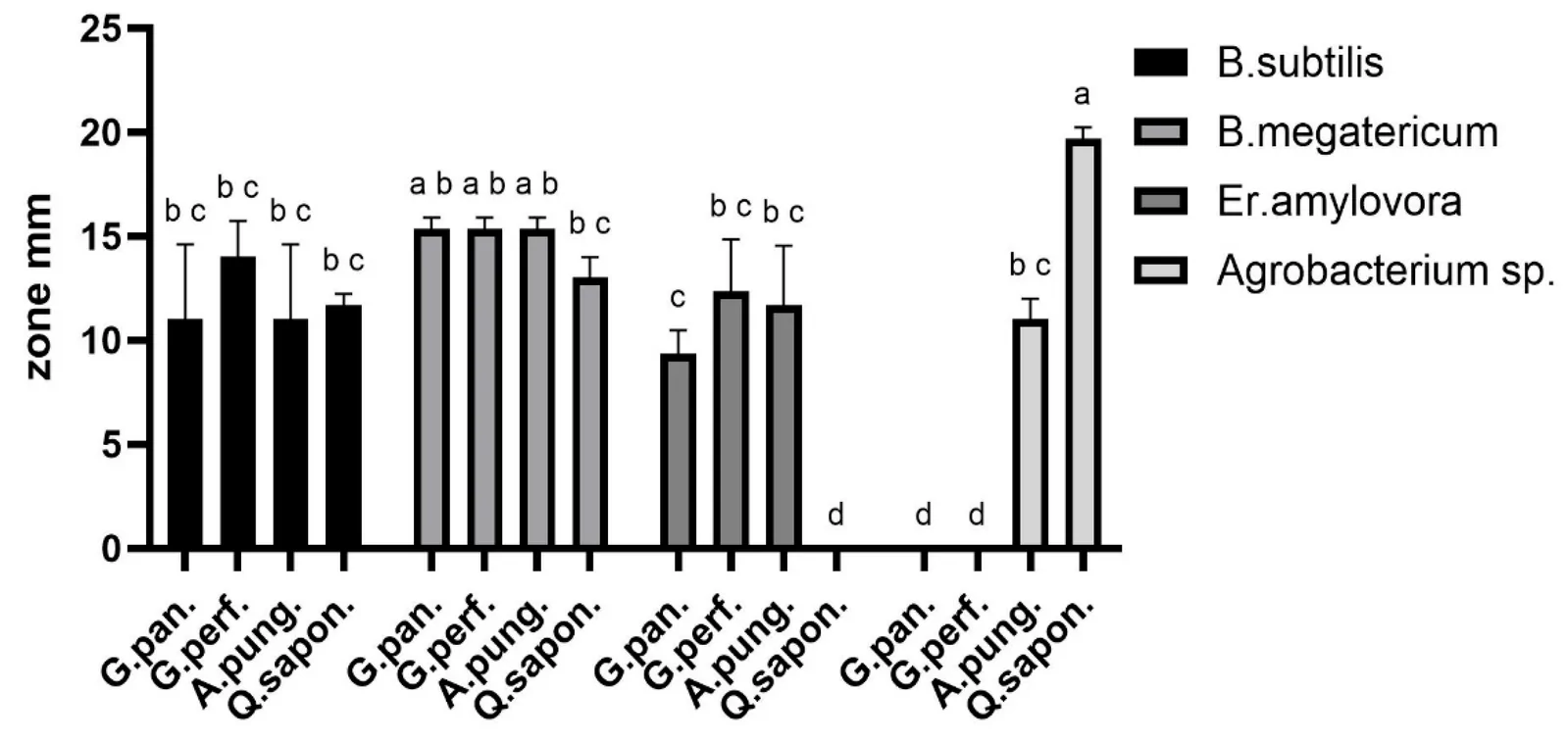
Growth-inhibiting activity of total ethanol extracts of *Gypsophila paniculata*, *G. perfoliata*, and *Acanthophyllum pungens*, and commercial saponins preparation *Quillaja saponaria* at dose of 250 µL on the test bacterial cultures.

A comparison of the data showed that the antibacterial effect of studied extracts at 100 µL and 250 µL, as measured by the *Bacillus* inhibition zone, did not differ significantly from that of the commercial *Q. saponaria* preparation (Figure 6).

The effect of *A. pungens* extract was nearly half as effective against *Agrobacterium* sp. At the same time, the *Q. saponaria* preparation did not inhibit the growth of the *Er. amylovora*, unlike the experimental extracts. The antifungal activity of the *Gypsophila* extracts were significantly lower than that of the comparative control. The inhibitory effect of *A. pungens* against *F. solani* and *A. niger* was comparable to that of the *Q. saponaria*, but was significantly inferior to it against *F. graminearum*.

## 3. Discussion

Previous studies on the saponin-bearing species *Acanthophyllum gypsophiloides* (*Allochrusa gypsophiloides*) have established a relationship between saponins accumulation and root morphometry, plant age, and ontogenetic stage [29]. A bell-shaped relationship was found between the amount of saponins in the roots and the age of *A. gypsophiloides* plants. The highest saponin content was found in medium-diameter roots from generative, middle-aged plants. Young roots, which were 2–3 times smaller in diameter, contained no more than 4% saponins, and very large roots from older plants also exhibited a decrease in concentration due to age-related changes in the tissues. The saponin content increased with the age of the plants until it reached a certain plateau [29]. Similar results were previously obtained when the species was introduced in Turkmenistan, where the highest accumulation of saponins was observed in the fifth year of cultivation [33,34]. A dependence of saponin accumulation in *A. gypsophiloides* on the ontogenetic phase was identified: in the roots, the content increases from the beginning of the growing season to the flowering–fruiting phase; in the aboveground parts—the maximum level reached during the active growth and flowering phase decreases during the fruiting stage, reflecting the redistribution of secondary metabolites between the aboveground and underground parts of the plant [30]. Studies on *A. gypsophiloides* have shown that the most informative indicators for harvesting high-quality raw materials and standardizing medicinal plant material are the diameter of the taproot and the age of the plant. For the genus *Gypsophila*, there are virtually no studies linking saponin content to root morphometry or data on their seasonal dynamics, which represents a significant gap [34]. In the study by Kolodziej et al. conducted for three species of *Gypsophila*, harvesting after at least three years of cultivation for production of raw material with high saponin content was recommended [28]. A comparative assessment conducted in this study of root parameters and saponins content depending on the timing of root harvesting at different growth stages of wild species *G. paniculata*, *G. perfoliata*, and *A. pungens*, which grow in southeastern Kazakhstan, revealed similar patterns and certain differences among the species. It was found that, for all species, the variability of key root parameters within a single harvest differed across various stages of plant growth. For example, regarding root diameter, *G. paniculata* samples exhibited a significant coefficient of variation (CV) of 40.23% during the flowering stage; in the other growth stages, this value ranged from 20% to 28%, which is entirely acceptable for field studies. For the *A. pungens* and *G. perfoliata* samples, significant differences in root diameter were observed among samples during the vegetative stage (CV 48.83% and CV 46.57%, respectively). High variability was observed in the fresh and dry weights of roots across all species within each harvest. At the same time, weight parameters for all species showed a significant positive correlation with root diameter. It is known that root system development is closely linked to the phases of the growing season: during the vegetative and flowering stages, the root increases in length, diameter, and weight; at the end of the growing season, as the plant enters dormancy, root growth slows down, and the plant begins to accumulate reserve substances [35]. The varying degrees of variability in root parameters among species may be related to differences in the morphology and developmental characteristics of their root systems. The *G. paniculata* and *G. perfoliata* root systems featured a distinct, thickened, cylindrical taproot from which longitudinally oriented lateral roots of varying lengths extended. In contrast, the *A. pungens* root system was characterized by a shortened, poorly defined taproot and numerous lateral roots oriented both longitudinally and horizontally. It should be noted that the root morphology for these species, as well as their seasonal development, require separate detailed study.

When comparing the average data of each harvest for each species separately, certain differences were identified. It was found that *G. paniculata* samples from different harvests did differ in root diameter with a 95% confidence level. A similar pattern was observed for *G. perfoliata* samples. For the species *A. pungens*, samples varied significantly in fresh and dry weight both within a single collection and between different collections, but the differences in root diameter were less significant. Based on these data, it can be assumed that during the growing season, the populations of *G. paniculata* and *G. perfoliata* remained consistent in terms of weight but varied slightly in root diameter. This may indicate minimal age-related heterogeneity within these populations. In contrast, the *A. pungens* population exhibited high variability in growth parameters throughout the growing season, which may be due to differences in the population’s age structure and the species’ natural intrapopulation heterogeneity. Interspecific differences may be related to both genetic differences between *Gypsophila* and *Acanthophyllum* and to differences in the phenotypic heterogeneity of the populations.

The literature review has shown that root development is closely associated with saponin accumulation. In *Achyranthes bidentata*, increases in root length and diameter were accompanied by a comparable increase in triterpenoid saponin accumulation, while structural differentiation of the root was associated with changes in the localization and accumulation of these compounds [36]. A comparable relationship between root structural development, biomass and saponin accumulation was reported for *Polygala tenuifolia* [37]. Seasonal patterns and developmental changes in saponin content in the roots were also demonstrated for *Panax notoginseng*; characteristic temporal patterns of accumulation were observed for individual saponins [38].

In our study the nature of the relationship between root growth parameters and saponin content and the growth stages of wild plants varied depending on the species. Correlation analysis of data from all *G. perfoliata* collections revealed a moderate positive correlation between saponin content and root diameter; however, no statistically significant differences in saponin content were found among samples across the different growth stages. In *G. paniculata*, which exhibited similar root morphometric parameters across growth stages, the highest saponin levels were observed in samples isolated during the flowering stage from plants with the smallest root diameter. Correlation analysis of all data also revealed a significant negative correlation between saponin content and root diameter in *G. paniculata* samples (r = – 0.47). In *A. pungens* samples, high saponin levels were observed only toward the end of the growing season, when the roots reached their maximum size. Saponin levels correlated moderately with the dry weight of the *A. pungens* raw material. The differences observed between *Gypsophila* and *A. pungens* across various growth stages indicate genotypic variations between them. It was found that at the beginning of the growing season, the species did not differ in saponin content; interspecific differences in saponin levels were observed during the flowering stage and at the end of the growing season. The saponin content in the roots of *A. pungens* increased significantly—by a factor of two—by the end of the growing season, whereas in *G. paniculata*, the maximum saponin level in the roots was observed during the flowering.

A limitation of the present study is that the number and timing of collections were not identical among the studied species. Sampling was performed according to the phenological development and availability of plant material for each species, resulting in differences in sampling dates and in the number of collections. Therefore, direct quantitative comparisons of saponins level among species should be interpreted with caution, because the observed differences may reflect both species-specific characteristics and variation associated with phenological stage. Nevertheless, the sampling design allowed us to characterize the seasonal dynamics of saponin accumulation within each species and to identify species-specific relationships between saponin levels and the root growth during the plant growing season. Further studies based on synchronized sampling at equivalent phenological stages and with balanced numbers of biological replicates would be required for rigorous interspecific comparisons.

The antimicrobial activity of saponin extracts has been identified in many studies and described in reviews [8,9,16,17]. Triterpene saponins isolated from *Sapindus mukorossi* pulp extracts exhibited growth-inhibiting effects against *Trichophyton rubrum* and *C. albicans* with an MIC 80 value of 8 μg/mL [39]. The extracts of saponin from the roots of *Glycyrrhiza glabra* and *Quillaja saponaria* show in vitro antifungal activity against *C. albicans* and *C. tropicalic*, with an inhibition zone of 15.3 and 16.5 mm, at a dose of 20 mg/mL [40].

The current literature on the species under study consists mainly of chemical studies of the composition and structure of saponins and studies of the biological activity of extracts from plant material of the genus *Gypsophila*. However, data on the antimicrobial activity of the genus *Acanthophyllum* are extremely limited. The authors of a recent review covering more than 150 species of *Gypsophila* note that antimicrobial activity is one of the most promising areas of research on this genus [2]. In studies by Kolodziev et al. involving three species of *Gypsophila*, a direct correlation was found between saponin content and the antibacterial activity of the extracts against G+ *Staphylococcus aureus* and *Bacillus subtilis* [28]. Extracts of *Gypsophila eriocalyx* also effectively inhibited the growth of *Staphylococcus aureus* and *Bacillus cereus*. The authors of the studies suggest that the growth-inhibiting activity is due not only to saponins but also to synergism with phenolic compounds and flavonoids [41]. Petroleum ether and ethyl acetate extracts of *G. pilosa* and methanol extracts of *G. perfoliata* were formed high inhibition zone against bacteria *Proteus vulgaris* [42].

Dozens of individual triterpene saponins, including acanthophyllosides, allochrosides, and quillaeic acid derivatives, have been identified in the roots of the endemic species *Acanthophyllum* (*Allochrusa gypsophiloides*) [34,43]. Recent studies have shown that both wild-growing plants and in vitro cultures of A. *gypsophiloides* retain high saponin content and exhibit antibacterial activity primarily against G+ microorganisms [44]. Recent studies on *Acanthophyllum elatius* have demonstrated antibacterial activity against *Escherichia coli*, *Staphylococcus aureus*, and *Bacillus subtilis* by saponin fractions isolated using ultrasonic extraction combined with LC-ESI-MS profiling, confirming the promise of further research on this genus [45]. A common pattern observed in the cited studies is the noted greater efficacy of saponin extracts against G+ bacteria compared to G– microorganisms, which is explained by the presence in the latter of an outer lipopolysaccharide membrane that prevents the penetration of large amphiphilic saponin molecules. All studies report a positive correlation between saponin content and the strength of the antimicrobial effect; however, the strength of this correlation varies significantly and likely depends on the structure of individual saponins and their interaction with other secondary metabolites [9,17]. Despite the structural diversity of saponins, most studies indicate that their antimicrobial mechanisms of action are similar [23].

The extracts obtained from *Gypsophila* and *A. pungens* differed in their antibacterial and antifungal activities. The growth-inhibiting activity of *Gypsophila* extracts against G+ bacteria was slightly higher compared to G– ones. *Gypsophila* species exhibited a similar inhibitory effect against G+ *Bacillus* and G– *Er. amylovora*, but were ineffective against G– *Agrobacterium* sp. The antifungal activity of the two *Gypsophila* species against *Fusarium* was maximal at the minimum effective dose. This may be due to the presence of other secondary compounds in the total extract that reduce the inhibitory effect or by their interaction with saponins. The growth-inhibitory activity of *A. pungens* against *Bacillus* and *Er. amylovora* did not vary significantly among the test bacterial species. However, unlike *Gypsophila* species, *A. pungens* exhibited dose-dependent growth-inhibitory activity against G– *Agrobacterium* sp. The antifungal activity of *A. pungens* against the *A. niger* was also dose-dependent, with a large inhibition zone observed at the maximum extract dose. The result of statistical analysis demonstrated that the antibacterial activity of saponin extracts of these species depends on the target pathogen bacteria and extract dose selected for each test-culture. The strength of antifungal activity of all extracts against *Fusarium* and *Aspergillus niger* is determined primarily by the extract apply dose.

## 4. Materials and Methods

### 4.1. Collection of Plant Material

The source plant material of the wild species *Gypsophila paniculata* L., *Gypsophila perfoliata* L. and *Acanthophyllum pungens* (*Bunge*) *Boiss*., was collected from natural populations in the desert region of southeastern Kazakhstan within the Almaty Region. Populations of *G. perfoliata* were identified in the Ili River valley and the Taukum Sands in the Southern Pribalhash region at elevations ranging from 406 to 416 m above sea level Populations of *G. paniculata* were found on the semi-fixed Taukum sands, on the tops and at the bases of the sand dunes, at approximately the same elevation as *G. perfoliata*—from 405 to 419 m above sea level. Stands of *A. pungens* grew on the foothill plain of Mount Bala Bogety and in the northeastern and eastern spurs of the Zailiyskiy Alatau at elevations ranging from 553 to 564 m above sea level (Figure 1).

### 4.2. Preparation of Total Saponins Extracts

To obtain total ethanol extracts, standard methods were used with some modifications [46]. Dry plant material weighing 2–5 g was first purified of unwanted impurities using chloroform, followed by extraction with 70° ethanol (1:40) for 2 h in a Soxhlet apparatus; the ethanol extracts were then evaporated under vacuum to a dry residue, and the weight of the resulting total extract was determined. The extracts were freeze-dried and stored at −20 °C until analysis.

Roots were excavated from representative specimens of *G. paniculata*, *G. perfoliata*, and *A. pungens* between April–May and September at various stages of their growth. Plant material from the studied species was identified at the Institute of Botany and Phytointroduction of the Ministry of Ecology and Natural Resources of the Republic of Kazakhstan, and herbarium specimens were preserved. Root growth parameters (average diameter and fresh weight) were determined in the field immediately after excavation. The moisture content of the freshly collected material was determined using the moisture analyzer (Evalas, Novosibirsk, Russia) in triplicate. The root samples were then air-dried naturally, and their dry weight was determined. The material was stored in parchment bags at room temperature and 40% humidity until analysis. The sample group from each collection included root material from 4 to 10 plants at the same stage of vegetation.

### 4.3. Determination of Total Saponin Content in Extracts

The total saponin content (TS) in the extracts was determined using a spectrophotometric microplate method based on the formation of a colored complex upon the interaction of saponins with vanillin in the presence of concentrated sulfuric acid [47,48]. To 25 μL of the sample, 25 μL of an 8% vanillin (Sigma-Aldrich, Darmstadt, Germany) solution and 250 μL of 72% sulfuric acid were added. The microplate containing the reaction mixture was incubated at 60 °C for 15 min with stirring, then cooled in an ice bath to room temperature. The optical density of the solutions was measured at λ 510–540 nm (Multiskan SkyHigh, Life Technologies Holdings Pte Ltd., Singapore), and the results, normalized to the quillic acid from *Quillaja saponins* (Sigma-Aldrich, Bangalore, India) as a reference standard (concentration curve in the range of 0.01–0.12 mg/mL), were expressed as a percentage of absolutely dry raw material using the following formula:

*TS* = *C* × *V*_t_ × *D* × 10^−3^ × 100 × 100/*V*_al_ × *m*_s_ × (100 − *M*) (1)

where TS—total saponin content (% a.d.w.), C—concentration from the curve, (mg/mL), V_t_—total volume of extract (ml), V_al_—volume of aliquot (ml), D—dilution factor, m_s_—the mass of sample (g), 10^−3^—conversion factor for mg in g, 100—%, M—moisture %.

### 4.4. Antimicrobial Activity of Total Extracts

The antimicrobial activity of total extracts was determined by the agar diffusion method using paper discs [49] at the “Research and Production Center of Microbiology and Virology” of the Ministry of Science and Higher Education of the Republic of Kazakhstan (Almaty). Collection strains of conditionally pathogenic microorganisms, including the bacteria *Bacillus subtilis*, *Bacillus megatericum*, *Erwinia amylovora*, and *Agrobacterium* sp., and the fungi *Fusarium solani*, *Fusarium graminearum*, and Aspergillus niger, were used as test cultures. An initial assessment was conducted to determine the antibiotic susceptibility of the test cultures to lincomycin (20 μL), ampicillin (10 μL), erythromycin (15 μL), and nystatin at a dose of 100 μL. The test extracts were dissolved in 70° ethanol at a 10:1 ratio and applied to discs containing 25 μL, 50 μL, 100 μL, and 250 μL, corresponding to 0.25 mg, 0.5 mg, 1.0 mg, and 2.5 mg of the original extract, respectively. The treated discs were air-dried to remove ethanol before testing. On the surface of the culture medium—fish peptone agar (FPA), sugar peptone agar (SPA), or Yemenn medium (for bacteria) or Čapek medium (for fungi) using a Drigalski spatula (Neolab, Heidelberg-Wieblingen, Germany), and discs impregnated with the extracts were placed on them. The resulting plates were incubated in a thermostat for 24 h at 37 °C. A 20-μL dose of 70° ethanol served as a negative control. A commercial saponins derived from Quillaja bark was used as the control at concentrations of 50 μL, 100 μL, and 250 μL per disk (10:1 stock solution). Antagonistic activity was assessed based on the diameter of the zones of pathogen growth inhibition formed around the discs, measured using a template ruler for measuring inhibition zones.

### 4.5. Statistical Analyses

All calculations and graphical representations were performed using GrapPad Prizm version 10.6.1 (892) for Windows, GrapPad Software, Boston, MA, USA (www.graphpad.com/ accessed on 8 June 2026). The one-way and two-way analysis (ANOVA) followed by Tukey’s multiple comparisons test was performed using spreadsheet software tools, version 10.6.1 (892. A correlation matrix was constructed and visualized as a heatmap to facilitate interpretation of relationships between variables. Statistically significant correlations were determined based on Pearson’s correlation coefficients with corresponding *p*-values. Significance levels were indicated as follows: * *p* < 0.05, ** *p* < 0.01, and *** *p* < 0.001. In the experiments on the quantitative analysis of saponins, the number of biological replicates corresponded to the number of independently sampled individual roots for each species and collection date. For each collection date, individual roots were treated as independent biological replicates and were analyzed separately, plant material from different individuals was not pooled. Microbiological tests for each dose of the extract were conducted in triplicate. In the one-way ANOVA, only those extract doses that produced a nonzero effect were included, inactive doses (no activity) were excluded from the analysis. In the two-way ANOVA, the absence of activity was treated as a zero value and such data were included in the analysis. A two-factor ANOVA was performed for all extracts using four bacterial test strains and four doses (25 µL, 50 µL, 100 µL, 250 µL), as well as separately for three fungal cultures using all the specified doses.

## 5. Conclusions

Thus, the patterns of saponin accumulation in the roots of *G. paniculata*, *G. perfoliata*, and *A. pungens* were identified based on their morphometric parameters and the vegetative phase of the plants. The differences observed among the species indicate varying seasonal dynamics of root growth, which influence the accumulation of saponins in the roots. The observed intraspecific and interspecific variability in morphometry and TS among these species may be associated with intrapopulation heterogeneity, as well as with differences in the plants’ growth conditions. The results obtained indicate that *Gypsophila* and *Acanthophyllum* show promise as sources of triterpene saponins with growth-inhibiting activity for further study.

## Figures and Tables

**Table 1:** Collection dates for plant raw materials and growth stages of the wild species *G. paniculata*, *G. perfoliata*, and *A. pungens* growing in the Almaty Region.

Species	Phase of Vegetation and Collection Dates			
	Vegetation	Budding	Flowering—Fruiting	The End of Vegetation
*G. paniculata*	15 April	14 May	20 May	11 June, 2 July, 6 August, 5 September
*G. perfoliata*	5 May, 12 June	13 July	7 August	8 September
*A. pungens*	6 May		29 May	13 June, 8 August, 10 September

**Table 2:** Average data on diameter (D), fresh weight (FW) and dry weight (DW), moisture (M) and weight loss (WL) of root samples of studied species.

Root Parameter	*G. paniculata*	*G. perfoliata*	*A. pungens*	ANOVA
D	a 3.26 ± 0.14 ***	b 2.10 ± 0.19 ***	b 1.87 ± 0.10 ***	(2, 122) = 29.62 ***
FW	a 0.49 ± 0.04 ***	b 0.10 ± 0.01 ***	b 0.10 ± 0.01 ***	(2, 122) = 67.37 ***
DW	a 0.23 ± 0.02 ***	b 0.05 ± 0.01 ***	b 0.07 ± 0.01 ***	(2, 122) = 45.87 ***
M	a 55.27 ± 3.45 ***	ab 47.03 ± 4.08 ***	b 35.34 ± 1.90 ***	(2, 36) = 9.50 ***
WL	a 58.13 ± 1.52 ***	a 53.58 ± 2.83 ***	b 37.65 ± 2.24 ***	(2, 120) = 26.37 ***

D cm, FW and DW kg, M and WL %, *** *p* < 0.001: very highly significant; a, b: statistically significant differences between data points in the same row.

**Table 3:** Results of testing test strains of microorganisms against standard antibiotic controls.

Test Culture	Inhibition Zone, mm			
	LCM (20 µg)	AMP (10 µg)	ETM (15 µg)	NYT (100 µg)
*Bacillus megatericum*	14.66 ± 0.57	2.00	11.66 ± 1.15	-
*Bacillus subtilis*	9.33 ± 1.15	15.33 ± 0.57	10.00±	-
*Erwinia amylovora*	16.00±	15.00±	20.00±	-
*Agrobacterium* sp.	9.66 ± 0.57	15.00	16.0 ± 1.73	-
*Fusarium solani*				NA
*Aspergillus niger*				10.66 ± 1.15
*Fusarium graminearum*				14.33 ± 1.15

LCM, lincomycin; AMP, ampicillin; ETM, erythromycin; NYT, nystatin; NA, no activity.

**Table 4:** Growth-inhibiting activity of total ethanol extracts of *Gypsophila paniculata*, *G. perfoliata*, and *Acanthophyllum pungens* on the test bacterial cultures.

	Test-Culture (Gram Reaction) and Inhibition Zone (mm)				
Dose (µL)	*Bacillus subtilis* (+)	*B. megatericum* (+)	*Erwinia amylovora* (−)	*Agrobacterium* sp. (−)	ANOVA
Control Ethanol 70%, 20	NA	NA	5.66 ± 1.15	2.33 ± 0.57	
*Q. saponaria* (10:1) 250	11.67 ± 0.33 *** B	13.0 ± 0.58 ** B	NA	19.67 ± 0.33 *** A	F (2, 6) = 99.20 ***
Extract					
*G. paniculata* (10:1)					
25	NA	NA	b 2.67 ± 0.33 *	NA	
50	NA	NA	a 9.00 ± 1.00 *	NA	
100	a 14.67 ± 1.45 ** A	a 14.67 ± 0.33 *** A	b 4.67 ± 0.58 ** B	NA	F (2, 6) = 42.86 ***
250	b 11.00 ± 2.08 * AB	a 15.33 ± 0.33 *** A	a 9.33 ± 0.67 ** B	NA	F (2, 6) = 5.89 *
ANOVA	F (3, 8) = 2.33 ^ns^		F (3, 8) = 25.84 ***		
*G. perfoliata* (10:1)					
25	NA	NA	c 4.00 ± 0.58 *	NA	
50	NA	NA	b 9.33 ± 0.67 **	NA	
100	a 14.33 ± 0.67 ** A	a 15.00 ± 1.15 ** A	c 4.00 ± 1.00 ^ns^ B	NA	F (2, 6) = 41.08 ***
250	a 14.00 ± 1.00 ** A	a 15.33 ± 0.33 ** A	a 12.33 ± 1.45 * A	NA	F (2, 6) = 2.10 ^ns^
ANOVA	F (3, 8) = 0.51 ^ns^		F (3, 8) = 13.16 **		
*A. pungens* (10:1)					
25	NA	NA	NA	b 5.33 ± 0.33 **	
50	NA	NA	NA	a 11.33 ± 0.67 **	
100	a 14.67 ± 1.45 ** A	a 14.67 ± 0.33 *** A	NA	b 4.67 ± 0.33 ** B	F (2, 6) = 42.86 ***
250	b 11.00 ± 2.08 * A	a 15.33 ± 0.33 *** A	11.67 ± 1.67 * A	a 11.00 ± 0.57 ** A	F (3, 8) = 2.29 ^ns^
ANOVA	F (4, 10) = 2.08 ^ns^			F (3, 8) = 51.07 ***	

NA no activity, ^ns^ no significant; * *p* ≤ 0.05 statistically significant; ** *p* ≤ 0.01 highly significant; *** *p* ≤ 0.001 very highly significant; a, b, c: statistically significant differences between data points in the column for each species, A, B: statistically significant differences between data points in the row.

**Table 5:** Growth-inhibiting activity of total ethanol extracts of *Gypsophila paniculata*, *G. perfoliata*, and *Acanthophyllum pungens* on the test fungal cultures.

	Test-Culture and Inhibition Zone (mm)			
Dose (µL)	*Fusarium solani*	*Aspergillus niger*	*F. graminearum*	ANOVA
Control				
Ethanol 70%				
20	NA	NA	NA	
*Q. saponaria* (10:1)				
250	10.67 ± 0.67 ** B	10.07 ± 0.07 *** B	15.10 ± 0.10 *** A	F (2, 6) = 49.41 ***
Extract				
*G. paniculata* (10:1)				
25	NA	NA	NA	
50	a 8.00 ± 1.00 * A	a 4.33 ± 0.67 * A	a 7.67 ± 2.33 ^ns^ A	F (2, 6) = 1.79 ^ns^
100	a 7.33 ± 1.45 * A	a 1.83 ± 0.17 ** B	a 4.33 ± 0.67 * AB	F (2, 6) = 8.81 *
250	a 3.68 ± 0.67 *	NA	a 5.67 ± 0.67 *	
ANOVA	F (2, 6) = 4.59 ^ns^		F (2, 6) = 1.33 ^ns^	
*G. perfoliata* (10:1)				
25	NA	NA	NA	
50	a 9.33 ± 0.67 ** A	6.67 ± 0.33 ** B	a 7.67 ± 0.33 ** AB	F (2, 6) = 8.17 *
100	b 2.67 ± 0.33 *	NA	NA	
250	b 3.67 ± 0.67 *	NA	b 4.33 ± 0.33 **	
ANOVA	F (2, 6) = 38.78 ***			
*A. pungens* (10:1)				
25	NA	c 1.93 ± 0.07 **	NA	
50	b 4.67 ± 0.33 ** B	b 5.07 ± 0.07 *** B	a 8.33 ± 0.88 * A	F (2, 6) = 13.59 **
100	NA	NA	NA	
250	a 10.00 ± 1.16 * A	a 9.67 ± 1.45 * A	a 8.67 ± 0.67 ** A	F (2, 6) = 0.37 ^ns^
ANOVA		F (2, 6) = 21.41 **		

NA no activity, ^ns^ no significant; * *p* ≤ 0.05: statistically significant; ** *p* ≤ 0.01: highly significant; *** *p* ≤ 0.001: very highly significant; a, b: statistically significant differences between data points in the same column for each species; A, B: statistically significant differences between data points in the row.

## Data Availability

The original contribution presented in this study are included in the article. Further inquiries can be directed to the corresponding author.

## Abbreviations

The following abbreviations are used in this manuscript:

TS	Total saponin content
a.d.w.	Absolutely dry weight
D	Diameter of root
FW	Fresh weight or root
DW	Dry weight of root
M	Moisture of root

## Appendix A

**Table A1:** Diameter, fresh weight, dry weight, moisture of root of *G. paniculata*, *G. perfoliata*, and *A. pungens* and total saponin content (TS) in the root samples collected from natural populations during growing season in the Almaty Region.

Collection	Phase	Samp.	Diameter		Fresh Weight		Dry Weight		Moisture		TS	
			D cm	CV %	kg	CV %	kg	CV %	%	CV %	% a.d.w.	CV %
*G. paniculata*												
14 April	V	6	ab 3.50 ± 0.34 ***	23.90	a 0.39 ± 0.1 **	60.67	b 0.13 ± 0.03 **	58.49	b 48.07 ± 1.78 *	5.22	c 31.87 ± 4.59 ***	35.30
14 May	B	5	ab 3.45 ± 0.38 ***	24.79	a 0.79 ± 0.21 *	57.98	ab 0.27 ± 0.09 *	74.69	ab 58.09 ± 5.44 ^ns^	13.23	bc 50.79 ± 8.58 **	37.75
20 May	F	6	b 2.28 ± 0.37 **	40.23	a 0.30 ± 0.06 **	48.64	b 0.10 ± 0.02 **	47.98	a 65.38 ± 1.16 *	2.51	a 98.35 ± 2.47 ***	6.16
11 June	EV	7	ab 3.43 ± 0.25 ***	19.16	a 0.44 ± 0.08 **	47.75	ab 0.19 ± 0.03 **	45.66	b 48.56 ± 0.81 *	2.36	c 40.59 ± 2.24 ***	14.62
2 July	EV	10	ab 3.27 ± 0.26 ***	25.27	a 0.51 ± 0.1 ***	56.94	a 0.34 ± 0.05 ***	46.11	a 63.77 ± 0.35 ***	0.96	bc 46.44 ± 5.15 ***	35.04
6 August	EV	5	ab 3.02 ± 0.27 ***	20.33	a 0.52 ± 0.05 ***	21.33	ab 0.21 ± 0.02 ***	20.19	a 67.07 ± 0.07 ***	0.18	b 69.84 ± 12.20 **	39.08
September	EV	4	a 4.15 ± 0.58 ***	27.97	a 0.48 ± 0.14 *	57.02	ab 0.24 ± 0.06 *	53.09	c 25.83 ± 1.29 *	7.04	c 30.48 ± 3.73 **	24.43
ANOVA (F, *p*)			(6, 36) = 2.41 *		(6, 36) = 1.81 ^ns^		(6, 36) = 3.44 **		(6, 9) = 56.81 ***		(6, 36) = 14.94 ***	
*G. perfoliata*												
5 May	V	10	a 3.13 ± 0.46 ***	46.57	a 0.12 ± 0.03 **	68.67	a 0.06 ± 0.01 **	69.33	a 60.20 ± 1.45 *	3.41	a 65.54 ± 15.24 **	73.52
12 June	V	7	b 1.54 ± 0.20 ***	33.60	a 0.09 ± 0.02 **	57.74	a 0.04 ± 0.01 ^ns^	108.8	a 54.06 ± 1.32 *	3.44	a 40.26 ± 4.14 ***	27.20
3 July	B	8	b1.56 ± 0.17 ***	31.59	a 0.11 ± 0.03 *	85.61	a 0.06 ± 0.03 ^ns^	126.9	b 43.29 ± 0.86 ***	3.43	a 39.69 ± 4.09 ***	29.14
7 August	F	6	b 1.48 ± 0.13 ***	21.28	a 0.08 ± 0.01 **	42.51	a 0.05 ± 0.02 **	39.62	a 56.20 ± 3.83 *	9.64	a 28.69 ± 4.81 **	41.06
September	EV	4	ab 2.50 ± 0.29 **	23.09	a 0.13 ± 0.03 *	47.48	a 0.04 ± 0.13 ^ns^	63.40	c 23.26 ± 1.29 *	7.82	a 48.97 ± 7.17 **	29.29
ANOVA (F, *p*)			(4, 30) = 5.67 **		(4, 30) = 0.60 ^ns^		(4, 30) = 0.34 ^ns^		(4, 6) = 59.91 ***		(4, 30) = 1.95 ^ns^	
*A. pungens*												
6 May	V	10	b 1.81 ± 0.28 ***	48.83	b 0.03 ± 0.01 ***	58.59	b 0.03 ± 0.003 ***	34.04	a 27.89 ± 0.95 *	4.79	b 36.87 ± 5.22 ***	44.74
29 May	F	11	b 1.50 ± 0.10 ***	22.46	b 0.10 ± 0.01 ***	56.25	b 0.05 ± 0.01 ***	51.40	a 43.36 ± 0.70 *	2.27	b 26.70 ± 2.48 ***	30.76
13 June	EV	8	b 1.71 ± 0.06 ***	10.56	b 0.10 ± 0.02 ***	48.01	b 0.06 ± 0.01 ***	36.79	a 32.83 ± 2.06 *	8.85	b 33.71 ± 3.08 **	25.83
8 August	EV	8	b 1.73 ± 0.12 ***	19.77	b 0.10 ± 0.01 ***	23.71	b 0.06 ± 0.01 ***	41.62	a 34.84 ± 1.13 *	4.59	b 19.23 ± 1.48 ***	21.80
September	EV	10	a 2.59 ± 0.26 ***	31.91	a 0.20 ± 0.03 ***	44.66	a 0.13 ± 0.02 ***	43.77	a 36.58 ± 4.12 **	22.55	a 65.28 ± 8.74 ***	42.33
ANOVA (F, *p*)			(4, 42) = 4.85 *		(4, 42) = 14.18 ***		(4, 42) = 14.04 ***		(4, 7) = 2.08 ^ns^		(4, 42) = 11.62 ***	

V, vegetation, B, budding, F, flowering, EV, end of vegetation, ^ns^ no significant,* *p* ≤ 0.05: statistically significant; ** *p* ≤ 0.01: highly significant; *** *p* ≤ 0.001: very highly significant; a, b: statistically significant differences in the same column, CV % coefficient variation.

**Table A2:** Total saponin content (TS) in roots of *G. paniculata*, *G. perfoliata*, and *A. pungens*, collected from natural populations at various stages of their growth in the Almaty Region.

Phase of Growth	*G. paniculata*	*G. perfoliata*	*A. pungens*	ANOVA
Vegetation	b 31.87 ± 4.59 *** A	a 55.13 ± 9.44 *** A	b 36.87 ± 5.22 *** A	(2, 30) = 1.89 ^ns^
Flowering	a 98.35 ± 2.47 *** A	a 34.98 ± 3.35 *** B	b 26.70 ± 1.32 *** B	(2, 28) = 106.8 ***
End of vegetation (September)	b 30.36 ± 3.62 ** B	a 48.20 ± 5.61 **AB	a 65.28 ± 8.74 *** A	(2, 16) = 3.84 *
ANOVA	(2, 18) = 11.32 ***	(2, 33) = 1.92 ^ns^	(2, 28) = 11.56 ***	

* *p* ≤ 0.05: statistically significant; ^ns^ no significant, ** *p* ≤ 0.01: highly significant; *** *p* ≤ 0.001: very highly significant; A, B: statistically significant differences between data points in the same row; a, b: statistically significant differences in the same column.

**Table A3:** Two-way analysis of variance and relative contributions of extract dose (25 µL, 50 µL, 100 µL, 250 µL), type of test bacterial culture (*Bacillus subtilis*, *B. megatericum*, *Erwinia amylovora*, *Agrobacterium* sp.), and their interaction to the growth inhibition zone diameter.

Extract	Factor	df	F	*p*	Significance	Contribution %
*G. paniculata*	Dose	3	(3, 32) = 138.00	≤0.001	***	36.76
	Test-culture	3	(3, 32) = 90.20	≤0.001	***	24.03
	Dose × test-culture	9	(9, 32) = 45.51	≤0.001	***	36.37
*G. perfoliata*	Dose	3	(3, 32) = 196.70	≤0.001	***	37.94
	Test-culture	3	(3, 32) = 128.30	≤0.001	***	24.76
	Dose × test-culture	9	(9, 32) = 60.88	≤0.001	***	35.24
*A. pungens*	Dose	3	(3, 32) = 157.10	≤0.001	***	49.45
	Test-culture	3	(3, 32) = 32.80	≤0.001	***	10.33
	Dose × test-culture	9	(9, 32) = 39.05	≤0.001	***	36.87

*** *p* ≤ 0.001: very highly significant.

**Table A4:** Two-way analysis of variance and relative contributions of extract dose (25 µL, 50 µL, 100 µL, 250 µL), type of test fungal culture (*Fusarium solani*, *F. graminearum*, *Aspergillus niger*) and their interaction to the growth inhibition zone diameter.

Extract	Factor	df	F	*p*	Significance	Contribution %
*G. paniculata*	Dose	3	(3, 24) = 27.11	≤0.001	***	53.67
	Test-culture	2	(2, 24) = 14.42	≤0.001	***	19.02
	Dose × test-culture	6	(6, 24) = 2.90	<0.05	*	11.47
*G. perfoliata*	Dose	3	(3, 24) = 336.5	≤0.001	***	83.65
	Test-culture	2	(2, 24) = 46.08	≤0.001	***	7.64
	Dose × test-culture	6	(6, 24) = 13.53	≤0.001	***	6.73
*A. pungens*	Dose	3	(3, 24) = 152.9	≤0.001	***	89.65
	Test-culture	2	(2, 24) = 0.99	>0.05	^ns^	0.39
	Dose × test-culture	6	(6, 24) = 4.49	≤0.01	**	5.27

^ns^ no significant,* *p* ≤ 0.05: statistically significant; ** *p* ≤ 0.01: highly significant; *** *p* ≤ 0.001: very highly significant.
